# Contribution of KIR3DL1/3DS1 to ankylosing spondylitis in human leukocyte antigen-B27 Caucasian populations

**DOI:** 10.1186/ar1988

**Published:** 2006-06-28

**Authors:** Carlos Lopez-Larrea, Miguel Angel Blanco-Gelaz, Juan Carlos Torre-Alonso, Jacome Bruges Armas, Beatriz Suarez-Alvarez, Laura Pruneda, Ana Rita Couto, Segundo Gonzalez, Antonio Lopez-Vázquez, Jesus Martinez-Borra

**Affiliations:** 1 Histocompatibility and Transplantation Unit, Hospital Universtario Central de Asturias, Celestino Villamil s/n. 33006 Oviedo, Asturias, Spain; 2 Rheumatology Unit, Hospital Monte Naranco, Avda Dres Fernandez Vega 107. 33012 Oviedo, Asturias, Spain; 3 Immunogenetic Service, Hospital de Santo Espirito de Angra do Heroismo, Vinha Brava. 9700 Angra do Heroismo, Azores, Portugal; 4 Functional Biology Department, University of Oviedo, Avda Julian Claveria s/n. 33006 Oviedo, Asturias, Spain

## Abstract

Killer cell immunoglobulin-like receptors (KIRs) and human leukocyte antigen (HLA) loci are both highly polymorphic, and some HLA class I molecules bind and trigger cell-surface receptors specified by *KIR *genes. We examined whether the combination of *KIR3DS1*/*3DL1 *genes in concert with HLA-B27 genotypes is associated with susceptibility to ankylosing spondylitis (AS). Two HLA-B27-positive Caucasian populations were selected, one from Spain (71 patients and 105 controls) and another from the Azores (Portugal) (55 patients and 75 controls). All were typed for HLA-B and *KIR *(*3DS1 *and *3DL1*) genes. Our results show that in addition to B27, the allele *3DS1 *is associated with AS compared with B27 controls (*p *< 0.0001 and *p *< 0.003 in the Spanish population and Azoreans, respectively). We also observed that the association of KIR3DS1 to AS was found in combination with HLA-B alleles carrying Bw4-I80 in trans position in the Spanish population (30.9% in AS versus 15.2% in B27 controls, *p *= 0.02, odds ratio (OR) = 2.49) and in Azoreans (27.2% in AS versus 8.7% in B27 controls, *p *= 0.01, OR = 4.4 in Azoreans). On the other hand, 3DL1 was decreased in patients compared with B27 controls (*p *< 0.0001 in the Spanish population and *p *< 0.003 in Azoreans). The presence of this allele in combination with Bw4-I80 had a protective effect against the development of AS in the Spanish population (19.7% in AS, 35.2% in B27 controls; *p *= 0.03, OR = 0.45). The presence of *KIR3DS1 *or *KIR3DL1 *in combination with HLA-B*27s/HLA-B Bw4-I80 genotypes may modulate the development of AS. The susceptibility to AS could be determined by the overall balance of activating and inhibitory composite *KIR*-HLA genotypes.

## Introduction

The association of ankylosing spondylitis (AS) with human leukocyte antigen (HLA)-B27 has been demonstrated worldwide, and evidence for the role of HLA-B27 in AS comes from linkage and association studies in humans and transgenic animal models. However, twin studies indicate that HLA-B27 contributes only 16% of the total genetic risk for disease [1]. Genome-wide scans have implicated regions on chromosomes 2q, 6p, 6q, 10q, 11q, 16q, 17q, and 19q in AS [2,3]. The killer immunoglobulin-like receptor (KIR) genes encode a group of proteins that are expressed on natural killer (NK) cells and in some T cells that are located on chromosome 19q13.4 in the leukocyte receptor complex (reviewed in [4]). KIR proteins act as receptors that recognise major histocompatibility complex (MHC) class I molecules and are directly involved in the activation and inhibition of NK and possibly also in CD8^+ ^T cells [5,6].

Given the receptor-ligand relationship between certain combinations of KIR and HLA class I molecules, it is reasonable to hypothesise a synergistic relationship between these polymorphic loci. This could be the case of the KIR3DL1 inhibitory receptor, the only KIR known to recognise HLA-B alleles. It binds to HLA-B with serological-defined epitope Bw4 (determined by amino acid positions 79–83 of the molecule) [7] with an isoleucine at position 80 (Bw4-I80) [8]. The interaction of KIR3DL1 and Bw4-I80 has an inhibitory effect on the cytotoxic capacity of NK cells. All HLA-B27 subtypes carry Bw4 epitope, with the exception of B*2708 and other related subtypes, which carry Bw6. From those with Bw4, B*2702 is the only subtype with an isoleucine at position 80 (Bw4-I80). The differences between the activating receptor KIR3DS1 and the inhibitory 3DL1 are located in the intracytoplasmic tail. The inhibitory receptor has a long tail containing immunoreceptor tyrosine inhibitory motifs, whereas the activating receptor has a short tail without this motif but with the capacity to interact with activating adaptor proteins such as DAP12 [9]. The ligand for KIR3DS1 has not been determined, although it has been shown that the KIR3DS1 activating receptor in combination with HLA-B alleles that encode molecules with isoleucine at position 80 (HLA-B Bw4-I80) results in delayed progression to AIDS after HIV-1 infection [10]. Recent studies have also reported a strong association of KIR/HLA combinations in the development of psoriatic arthritis [11].

The aim of this study was to analyse whether *KIR3DL1 *and *3DS1 *genes, and their possible synergistic effect with HLA-B alleles, influence the susceptibility to AS in HLA-B27-positive individuals.

## Materials and methods

### Patients and controls

Two B27-positive Caucasians populations were selected for this study, one from Spain (71 patients with AS and 105 healthy matched controls) and another from the Azores (Portugal) (55 patients with AS and 57 healthy matched controls). The patients with AS were diagnosed at the rheumatology units of the Hospital Universtario Central de Asturias and Hospital Naranco, Oviedo, Asturias, Spain, and the Rheumatic Diseases Clinic of the Angra do Heroismo Hospital, Azores, in accordance with New York criteria [12]. Radiographs of the pelvis and lumbar spine were obtained in all patients. Sacroiliac joint changes were determined on the basis of the New York criteria. All patients had sacroiliitis bilateral grade II or more. Both patients and controls gave written informed consent prior to enrolling in the study. The protocol was approved by the ethics committees of our hospitals and conducted according to the Declaration of Helsinki.

B27 subtypes were determined by polymerase chain reaction (PCR) using sequence-specific primer (SSP) as described previously [13]. HLA-B alleles and Bw4 and Bw6 epitopes were typed by PCR-sequence-specific oligoprobes (SSOs) with RELI™-SSO typing kits (Dynal Biotech, Oslo, Norway). *KIR3DL1 *and *3DS1 *genotyping was performed by PCR with gene-specific primer pairs in accordance with the method previously described [14]. Both were considered as alleles from the same locus.

Allelic frequencies were calculated by direct counting, and the significance of the association was determined using the χ^2 ^test with Yate's correction or Fisher's exact test. The odds ratio (OR) was calculated by the cross-product ratio. Exact confidence intervals of 95% were obtained. The χ^2 ^test was used for Hardy-Weinberg equilibrium (HWE) by comparing the observed number of subjects for each genotype with the expected number of subjects, assuming the existence of HWE.

## Results and Discussion

Here, we have analysed the possible influence of KIR genotypes in the susceptibility to AS in B27 individuals. The study was undertaken in two genetically distinct Caucasian populations but with a slightly different distribution of B27 alleles [15,16].

HLA-B27 represents a family of at least 27 closely related alleles (B*2701-27) that differ in their ethnic distribution. We used an SSP typing approach (PCR-SSP) capable of allelic detection of all B27 alleles described at present. The B27 alleles detected in our study can be classified into two categories: one group of alleles over-represented (B*2705, 02) and another group of alleles with minor representation such as B*2703, B*2707, B*2708, and B*2713. No differences were found in the distribution of B27 subtypes among patients with AS and B27-positive matched controls in either the Spanish or Azorean populations. We found some alleles that are present only in the Azorean population, such as B*2703 and B*2708, whereas we only found one individual with B*2713 in the Spanish population (Table 1).

However, differences in the distribution of the *KIR3DS1 *allele were found among these groups in both populations (Table 2). This allele was over-represented in the AS group compared with B27-positive healthy controls (42.9% versus 22.3%, *p *< 0.0001, OR = 2.6 in the Spanish population and 35.4% versus 17.5%, *p *< 0.003, OR = 2.58 in Azoreans). We also found a different distribution of the *KIR3DL1 *frequency among the groups because this gene and *KIR3DS1 *segregate as alleles. *KIR3DL1 *was found to be decreased in patients with AS compared with B27-positive controls (57% versus 77.6%, *p *< 0.0001, OR = 0.3 in the Spanish population and 64.5% versus 82.4%, *p *< 0.003, OR = 0.38 in Azoreans). Both alleles were also distributed differently when we considered the homozygous genotypes. *3DL1*/*3DL1 *was increased in B27-positive controls (*p *= 0.0008, OR = 0.33 in the Spanish population and *p *< 0.001, OR = 0.26 in Azoreans) compared with patients with AS. On the other hand, the *3DS1*/*3DS1 *genotype was increased in patients with AS compared with B27 controls, although in the Azorean population this was not significant with the size of the sample studied (*p *= 0.0009, OR = 3.75 in the Spanish population). When both genotypes are present (*3DS1*/*3DL1*), the activating effect appears to be prevailing and so this genotype is increased in patients in both populations, significantly in Azoreans (*p *= 0.005). No deviation from the HWE was detected in either patients or controls.

Finally, we analysed whether the Bw4-I80 ligand is involved in the susceptibility of *KIR3DS1 *to AS. We classified the HLA-B alleles of the subjects studied according to the presence of Bw4/Bw6 serological epitopes. The incidence of the B27 genotypes with HLA-B alleles in *trans* position with a Bw4-I80 (or in *cis* when the allele is B*2702) was not found to be different in patients with AS and both control groups. However, we found these two factors to be combined (3DS1/B27 genotypes with Bw4-I80 in trans) in 30.95% of patients with AS and in only 15.2% of B27-positive controls in the Spanish population (*p *= 0.02, OR = 2.49) and in 27.2% of patients with AS and in 8.7% of B27-positive controls in Azoreans (*p *= 0.01, OR = 4.4). Moreover, the inhibitory 3DL1/Bw4-I80 genotype was found to be increased in the control group compared with patients with AS in both populations, although not significantly in the Azorean population, possibly due to the size of the population.

Thus, the attributable effect of *KIR3DL1 *(protection) or *3DS1 *(susceptibility) on AS susceptibility may be more effective when the corresponding ligand Bw4-I80 is present and the effects could be additional to the presence of HLA-B*27. The presence of one single additional HLA-B allele carrying the Bw4-I80 epitope in B*27-positive individuals could modulate the state of cell activation of NK (and/or T) cells in conjunction with the pattern of *KIR *gene expression.

*KIR3DL1 *and *3DS1 *genes are polymorphic, and in the case of *KIR3DL1 *it has been described that this polymorphism may influence the expression [17] or the inhibitory effect [18] of the different alleles. This fact could also be important for the role of these genes in the susceptibility to AS.

The mechanism by which HLA-B27 confers susceptibility to inflammatory AS is not understood but is presumed to involve some unique aspect of its role in antigen presentation (reviewed in [19]). It has been argued that the T-cell response to an arthritogenic peptide(s) of endogenous origin and bound by HLA-B27 molecules might be the cause of spondyloarthropathies. In addition to their classical antigen-presenting role, it has been described that HLA-B27 are recognised by members of the KIR and leukocyte immunoglobulin-like receptor (ILT) families in both human and animal models. Members of KIR (3DL1 and 3DL2) and ILT (ILT4) are able to bind B27 in both classical β2m/heavy chain (HC) and β2m-free HC homodimers (HC-B27) that are dependent on the presence of Cys67 of the B27 molecule [20-22]. It has been argued that alternative recognition of different forms of HLA-B27 by KIR or ILT could influence their immunomodulatory function and may imply a role in inflammatory disease.

Binding of the KIR3DL1 receptor to the Bw4 family of MHC class I receptors has been shown to be dependent on key residues of the HC around isoleucine 80 [8]. However, it has been described that HLA-B*2705, which carries a Bw4 epitope containing threonine at residue 80, is also recognised by KIR3DL1, this interaction being peptide-specific [23]. In fact, it has been shown that KIR3DL1 recognises HLA-B27 molecules when loaded with a self-peptide and with a number of viral non-self-peptides. Nevertheless, some viral peptides such as EBV EBNA3C 258–266, which bind to HLA-B*2705, block the NK inhibitory receptor 3DL1 [24]. Anchor residues at position P7 and P8 of some viral peptides may prevent KIR3DL1-mediated recognition of HLA-B27. Similarly, it is probable that a significant fraction of B*27 'arthritogenic' ligands fail to interact with KIR3DL1 during the course of microbial infection, which thereby influences the activation of activating NK receptors (NKRs) (3DS1 and others) and subsequent progression to AS. It is therefore plausible that the presence of another HLA-B allele carrying the Bw4-I80 epitope may antagonise the unfavourable interaction of KIR3DL1 with an HLA-B27-peptide.

NKRs are expressed on subsets of effector and memory T cells [25]. It has previously been shown that stimulatory KIRs are able to promote proliferative responses of CD28^null ^T-cell clones, suggesting that the recognition of self-MHC class I molecules may lead to clonal expansion [26]. In a similar way to CD28, KIR receptors may function as T-cell receptor (TCR) co-stimulatory molecules. Interestingly, circulating effectors of CD4^+^CD28^null ^and CD8^+^CD28^null ^T cells were found to be expanded in AS and associated with more severe joint restrictions [27,28]. Expression of NKRs, including various members of the KIR family, was found on CD4^+^CD28^null ^T cells in patients with AS, and this resembles that found in rheumatoid arthritis [29,30].

The existence of a high degree of heterogeneity has been described in the expression of NKR by intrasynovial CD8^+ ^T cells that could modulate their cytotoxicity and play a role in the control of this HLA class I-associated autoimmune disease [31]. The activating KIR3DS1 in CD28^null^T cells could provide a mechanism through which TCR could be involved in enhancing and extending the immune response in AS, lowering the activation threshold for these cells, irrespective of their antigen specificity. The expression of KIR3DL1 may serve as a mechanism to control the activity of self-reactive T cells induced by arthritogenic peptides presented by β2m/HC or HC-B27, thus mediating T-cell tolerance to self-antigens. The genetic imbalance between KIR and their HLA class I ligands may enhance the activation of T cells with a low affinity for joint self-antigens, thereby contributing to the pathogenesis of AS.

## Conclusion

The presence of *KIR3DS1 *or *KIR3DL1 *in combination with HLA-B*27s/HLA-B Bw4-I80 genotypes may modulate the development of AS. The susceptibility to AS could be determined by the overall balance of activating and inhibitory composite *KIR*-HLA genotypes. Further analysis of NKR expression on T cells in patients with AS may help to elucidate the role of KIR receptor in predisposing to disease.

## Abbreviations

AS = ankylosing spondylitis; HC = heavy chain; HLA = human leukocyte antigen; HWE = Hardy-Weinberg equilibrium; ILT = immunoglobulin-like lymphocyte T receptors; KIR = killer cell immunoglobulin-like receptor; MHC = major histocompatibility complex; NK = natural killer; NKR = natural killer receptor; OR = odds ratio; PCR = polymerase chain reaction; SSO = sequence-specific oligoprobe; SSP = sequence-specific primer; TCR = T-cell receptor.

## Competing interests

The authors declare that they have no competing interests.

## Authors' contributions

CL conceived the study, participated in its design and coordination, and helped to draft the manuscript. JCT and JBA participated in the design of the study and in the selection of the patients. MAB, BS, LP, and ARC carried out the molecular genetic studies. SG participated in drafting the manuscript. AL participated in the design of the study and performed the statistical analysis. JM participated in the design of the study, carried out the molecular genetic studies, and participated in the statistical analysis. All authors read and approved the final manuscript.

## Figures and Tables

**Table 1:** Distribution of B27 subtypes in patients with ankylosing spondylitis (AS) and controls in Spanish and Azorian populations

	**Spanish**		**Azorian**	
	
Subtypes	**Patients with AS (*n *= 71)**	**Controls (*n *= 105)**	**Patients with AS (*n *= 55)**	**Controls (*n *= 57)**
B*2705	68 (95.7%)	94 (89.5%)	45 (81.8%)	52 (91.2%)
B*2702	3 (4.3%)	9 (8.6%)	7 (12.7%)	2 (3.5%)
B*2703	-	-	-	2 (3.5%)
B*2707	-	1 (0.95%)	-	1 (1.8%)
B*2708	-	-	3 (5.5%)	-
B*2713	-	1 (0.95%)	-	-

**Table 2:** Allelic and genotypic frequencies of KIR and HLA-B Bw4-I80 in patients with AS and B27 healthy controls in Spanish and Azorian populations.

Genotypes	**Spanish AS (2*n *= 142)**	**Spanish controls (2*n *= 210)**	***p***	**OR (95%CI)**	**Azorean AS (2*n *= 110)**	**Azorean controls (2*n *= 114)**	***p***	**OR (95%CI)**
*3DS1*	61 (42.9%)	47 (22.3%)	<0.0001	2.6 (1.64–4.15)	39 (35.4%)	20 (17.5%)	<0.003	2.58 (1.38–4.8)
*3DL1*	81 (57%)	163 (77.6%)	<0.0001	0.3 (0.24–0.6)	71 (64.5%)	94 (82.4%)	<0.003	0.38 (0.2–0.72)
Bw4I80	24 (16.9%)	40 (19%)	NS	-	18 (16.3%)	17 (14.9%)	NS	-
								
Genotypes	**Spanish AS (*n *= 71)**	**Spanish controls (*n *= 105)**			**Azorean AS (*n *= 55)**	**Azorean controls (*n *= 57)**		
*3DL1*/*3DL1*	25 (35.2%)	65 (61.9%)	0.0008	0.33 (0.17–0.62)	20 (36.3%)	39 (68.4%)	<0.001	0.26 (0.12–0.57)
*3DS1*/*3DS1*	15 (21.1%)	7 (6.6%)	0.0009	3.75 (1.44–9.74)	4 (7.2%)	2 (3.5%)	NS	-
*3DS1*/*3DL1*	31 (43.6%)	33 (31.4%)	NS	-	31 (56.1%)	16 (28%)	0.005	3.3 (1.50–7.26)
*3DS1*/Bw4I80 +	22 (30.9%)	16 (15.2%)	0.02	2.49 (1.2–5.19)	15 (27.2%)	5 (8.7%)	0.01	4.4 (1.48–13.37)
*3DS1*/Bw4I80 -	24 (33.8%)	24 (22.8%)	NS	-	20 (36.3%)	13 (22.8%)	NS	-
*3DL1*/Bw4I80 +	14 (19.7%)	37 (35.2%)	0.03	0.45 (0.22–0.91)	9 (16.3%)	16 (28%)	NS	-
*3DL1*/Bw4I80 -	42 (59.1%)	61 (58.1%)	NS	-	36 (64.4%)	39 (68.4%)	NS	-

AS, ankylosing spondylitis; CI, confidence interval; HLA, human leukocyte antigen; KIR, killer cell immunoglobulin-like receptor; NS, not significant; OR, odds ratio.
